# Supplementary material for: Synthesis of Organic Iodine Compounds in Sweetcorn under the Influence of Exogenous Foliar Application of Iodine and Vanadium
Source: Molecules. 2022 Mar 11;27(6):1822. doi: 10.3390/molecules27061822 (PMC8950039; doi:10.3390/molecules27061822)
Supplement: Supplementary file 1 [file molecules-27-01822-s001.zip › molecules-1597905-supplementary.pdf]

Table S1. Content of total sugars, ascorbic acid in grain and % dry weight from Experiment No.1 at 2018 and No.2 at 2019.

Treatment	Vitamin C [mg·100g $^{-1}$ d.w.]	Total Sugars mg·100g $^{-1}$ d.w.]	% D.W
		**Experiment No. 1_2018**	
Control	23.09 ± 1.06 ab	1448.31 ± 58.78 e	41.84 ± 1.38 a
V1	32.00 ± 0.58 def	1108.07 ± 17.11 c	40.51 ± 0.92 a
V2	21.06 ± 1.15 a	1146.36 ± 73.09 c	42.33 ± 1.07 a
KI	25.05 ± 0.39 abc	1211.36 ± 48.95 cd	39.46 ± 0.7 a
KI+ V1	26.75 ± 0.79 bcd	1399.23 ± 62.4 de	41.02 ± 0.73 a
KI +V2	29.86 ± 1.12 cde	1439.01 ± 58.1 e	40.05 ± 0.31 a
KIO_3	26.59 ± 0.76 bc	1074.44 ± 31.04 bc	42.65 ± 0.91 a
KIO_3 + V1	30.27 ± 1.57 cde	1285.62 ± 39.56 cde	39.93 ± 0.58 a
KIO_3 + V2	33.36 ± 1.06 ef	733.72 ± 28.58 a	42.65 ± 1.16 a
5ISA	25.59 ± 0.62 abc	817.7 ± 21.94 a	42.27 ± 0.53 a
5ISA + V1	29.6 ± 0.79 cde	793.22 ± 13.24 a	41.25 ± 0.59 a
5ISA + V2	28.59 ± 0.66 cde	1102.01 ± 41.03 c	43.07 ± 0.82 a
2IBA	35.74 ± 1.82 f	685.48 ± 18.9 a	40.44 ± 1.49 a
2IBA + V1	20.58 ± 0.3 a	858.96 ± 23.9 a	41.39 ± 0.68 a
2IBA + V2	42.93 ± 1.7 g	879.13 ± 35.14 ab	39.98 ± 1.42 a
Treatment		**Experiment No. 2_2019**	
Control	29.75 ± 0.36 a	660.71 ± 27.64 bcde	32.33 ± 0.19 de
V1	37.38 ± 0.48 bc	846.41 ± 13.93 gh	34.65 ± 0.18 e
V2	28.85 ± 1.8 a	875.16 ± 19.3 h	30.94 ± 0.74 cd
KI	39.24 ± 1.47 bc	828.28 ± 37.14 fgh	25.29 ± 1.06 a
KI+ V1	39.43 ± 1.22 bc	831.41 ± 18.27 fgh	27.83 ± 0.44 abc
KI +V2	38.52 ± 1.63 bc	705.79 ± 27.49 de	31.08 ± 0.69 cde
KIO_3	36.8 ± 0.56 b	551.25 ± 4.72 ab	30.77 ± 0.17 bcd
KIO_3 + V1	38.69 ± 1.58 bc	601.53 ± 38.66 abcd	31.33 ± 1.72 cde
KIO_3 + V2	50.15 ± 0.55 e	695.65 ± 13.57 cde	28.31 ± 0.45 abc
5ISA	38.97 ± 1.76 bc	871.99 ± 34.3 h	27.88 ± 1.01 abc
5ISA + V1	40.6 ± 0.84 bcd	755.69 ± 9.07 efg	30.05 ± 0.2 bcd
5ISA + V2	43.13 ± 0.91 cd	654.53 ± 16.26 bcde	28.55 ± 0.59 abc
2IBA	45.69 ± 0.75 de	493.00 ± 7.54 a	31.47 ± 0.48 cde
2IBA + V1	29.42 ± 1.08 a	590.54 ± 10.61 abc	29,00 ± 0.57 bcd
2IBA + V2	62.96 ± 1.41 f	721.66 ± 16.84 ef	27.18 ± 0.51 ab

Means followed by different letters for treatments differ significantly at $p < 0.05$ ($n = 8$). Bars indicate standard error.

Table S2. Content of total sugars, ascorbic acid in grain from and % dry weight Experiment No.3 at 2019/2020.

Treatment	Vitamin C mg·100g $^{-1}$ d.w.	Total sugars mg·100g $^{-1}$ d.w.	% D.W
Control	30.48 ± 3.75 bc	1862.48 ± 520.01 abc	30.69 ± 1.12 c
V1	29.91 ± 2.84 bc	2432.59 ± 711.32 e	28.13 ± 1.27 abc
KI	43.38 ± 4.62 d	2300.26 ± 576.33 de	25.51 ± 0.62 a
KI+ V1	33.48 ± 5.95 c	1713.01 ± 391.84 a	30.82 ± 1.06 c

KIO_3	48.61 ± 2.63 e	2157.15 ± 481.01 cde	26.89 ± 0.57 ab
KIO_3 + V1	27.75 ± 2.06 ab	2288.56 ± 629.38 de	28.99 ± 1.9 bc
5ISA	30.66 ± 1.13 bc	2288.2 ± 567.6 de	26.71 ± 0.7 ab
5ISA + V1	23.96 ± 1.12 a	1762.33 ± 404.77 ab	29.54 ± 1.33 bc
2IBA	28.49 ± 0.87 ab	1881.89 ± 393.65 abc	28.83 ± 0.75 bc
2IBeA + V1	29.45 ± 1.62 bc	2043.73 ± 521.47 bcd	30.26 ± 1.62 c

Means followed by different letters for treatments differ significantly at $p < 0.05$ ($n = 8$). Bars indicate standard error.
